# Mathematical Modeling of the HIV/Kaposi's Sarcoma Coinfection Dynamics in Areas of High HIV Prevalence

**DOI:** 10.1155/2013/753424

**Published:** 2013-11-17

**Authors:** E. Lungu, T. J. Massaro, E. Ndelwa, N. Ainea, S. Chibaya, N. J. Malunguza

**Affiliations:** ^1^Department of Mathematics, University of Botswana, Gaborone, Botswana; ^2^Department of Mathematics, University of Tennessee, TN, USA; ^3^Department of Mathematics, University of Dar es Salaam, Dar es Salaam, Tanzania; ^4^Department of Applied Mathematics, National University of Science and Technology, Bulawayo, Zimbabwe

## Abstract

We formulate a deterministic system of ordinary differential equations to quantify HAART treatment levels for patients co-infected with HIV and Kaposi's Sarcoma in a high HIV prevalence setting. A qualitative stability analysis of the equilibrium states is carried out and we find that the disease-free equilibrium is globally attracting whenever the reproductive number *ℛ *_*k*_ < 1. A unique endemic equilibrium exists and is locally stable whenever *ℛ *_*k*_ > 1. Therefore, reducing *ℛ *_*k*_ to below unity should be the goal for disease eradication. Provision of HAART is shown to provide dual benefit of reducing HIV spread and the risk of acquiring another fatal disease for HIV/AIDS patients. By providing treatment to 10% of the HIV population, about 87% of the AIDS population acquire protection against coinfection with HIV and Kaposi's Sarcoma (KS). Most sub-Sahara African countries already have programmes in place to screen HIV. Our recommendation is that these programmes should be expanded to include testing for HHV-8 and KS counseling.

## 1. Introduction

Kaposi's Sarcoma is a cancer that occurs mostly in humans with suppressed immune systems [1]. The development of this cancer depends upon prior infection to the human herpesvirus-8 (HHV-8) [2], a virus which is usually transmitted either sexually or via saliva [3]. For HIV-related Kaposi's Sarcoma development, immunosuppression is a necessary causal factor [2]. Because HIV is an immunosuppressive virus, it promotes the development of Kaposi's Sarcoma in individuals dually infected with both viruses (HIV and HHV-8) and this combination has proved to be fatal and has made Kaposi's Sarcoma the fourth largest killer of people living with HIV/AIDS in sub-Sahara Africa [4].

In competent immune systems, acquisition of HHV-8 does not guarantee the development of KS; in fact, most individuals with a strong immune response could remain latently infected with HHV-8 throughout their lifetime [2]. The HIV-1 growth factors stimulate the immune cells including the healthy and infected B-cells to proliferate. The activation of latently infected B-cells to proliferate only leads to production of more HHV-8 that, according to the theory proposed by Foreman et al. [2], may be responsible for infection of progenitor cells of endothelial origin, which once infected with HHV-8 develop into Kaposi's Sarcoma cells [2].

 Recent research findings [12] show that highly active antiretroviral therapy (HAART) for HIV significantly decreases (and, in some instances, completely forces the cancer into remission) KS activity in a patient, but such treatment is only effective against persons who have seroconverted. Lungu et al. studied the within-host dynamics of HIV and KS and came to the conclusion that HIV infection accentuates the potential for infected individuals to develop KS conditions and that administration of HAART on KS individuals who have seroconverted results in the reversal of KS conditions. However, the same result could not be demonstrated if HAART was administered to HIV negative individuals.

 From what has been described above, we formulate a mathematical model which includes the following classes: a susceptible class, *S*, two infected classes of individuals infected with HIV-1 only, *I*, individuals coinfected with HHV-8 and HIV-1, *I*_*k*_, two classes of asymptomatic infectives with HIV only, *P*, asymptomatic coinfected, *P*_*k*_, a class of treated individuals, *T*, and two AIDS classes, namely, individuals with full-blown AIDS only, *A*, and individuals coinfected with full-blown AIDS and Kaposi's Sarcoma. 

The structure of the paper is as follows. In Section 2 we formulate the model that describes the coinfection transmission dynamics. Existence of solutions is proved in Section 3, but our main interest is to simulate and study the dynamic behavior of the steady state solution. In particular, we want to quantify the proportion of HAART-treated individuals who never develop KS at various treatment rates *ϕ*_1_ and *ϕ*_2_.

 AIDS is the progressive stage at which an HIV-infected individual loses competency of his immune system and becomes prone to opportunistic infections. For this reason, HIV coinfection models have found a lot of space in HIV epidemiology studies. HIV and tuberculosis coinfection have been studied by Cohen et al. [13], Roeger et al. [14], and Ramkissoon et al. [15] while Barley et al. [9], Chiyaka et al. [16], and Mukandavire et al. [17] studied HIV-malaria coinfection dynamics with each study giving results pertinent to the coinfection under investigation. The study of HIV-KS coinfection dynamics is still in infancy and, to the best of our knowledge, no mathematical modeling study has been carried out to assess the coinfection dynamics of HIV and KS at the population level in sub-Sahara Africa. Because our model incorporates HAART administration to all infective classes, it can provide insights into treatment strategies and, in particular, decide whether the current policy based on a CD4 count threshold to access treatment should be continued as a strategy. 

## 2. Model Formulation

We begin with a human population of susceptibles which is free of both KS and HIV denoted by *S*. This population is replenished at constant rate Λ through sexual maturity or immigration. Upon effective contact with individuals infected with the HIV virus, the new infectives progress into the infected class *I* at rate *λ*_*h*_, where *λ*_*h*_ denotes the force of infection.

For simplicity of the model, we assume that all individuals in the class *S* are latently infected with the HHV-8 virus which causes the KS infection. Therefore, in our model every class, except for the susceptible class, is assumed to be at risk of developing KS. HIV infection is known to promote or enhance the development of KS condition [18, 19], and so we assume that individuals in the class *I* can develop mild KS at rate *ϵ*_1_ and move into the class of coinfected individuals KS denoted by *I*_*k*_. 

HIV-only and coinfected individuals are assumed to progress to the asymptomatic pre-AIDS classes (*P* and *P*_*k*_) at the same constant rate *ψ*_1_. Furthermore, persons in the pre-AIDS class are deemed to be sexually interacting and that individuals with mild KS in the *I*_*k*_ class can develop acute KS, which manifests in the form of visible lesions [19] and severe debilitation. Due to the nature of these symptoms, we assume that individuals who develop acute KS will no longer be sexually interacting. Those individuals in the *I*_*k*_ class, who develop acute KS, die at a disease induced rate *τ*_1_.

We assume that all infected individuals in the classes *I* and *I*_*k*_ are tested and if they are tested positive for HIV-1 they receive treatment at rate *ϕ*_1_ and move into the class of treated individuals, *T*. Individuals in the pre-AIDS classes *P* and *P*_*k*_ have higher viral loads and therefore possess less competent immune systems. These individuals will then present for medical attention at a higher rate *ϕ*_2_ > *ϕ*_1_. HAART is known to reverse KS conditions for people with HIV [19], and so in the model we assume that the therapy is perfect in reversing mild KS conditions. Upon accessing HAART, individuals in the infective class *I*_*k*_ and pre-AIDS class *P*_*k*_ achieve full KS recovery and progress to the treated class *T*. Additionally, individuals in the pre-AIDS class *P*_*k*_ progress to acute KS at rate *τ*_2_ > *τ*_1_. Individuals in the pre-AIDS class *P* are at risk of developing KS at a constant rate *ϵ*_2_. Individuals in the pre-AIDS classes *P* and *P*_*k*_ develop clinical symptoms and progress to full-blown AIDS *A* and *A*_*k*_, respectively, at rate *θ*_1_. Progression to full-blown AIDS by persons in the class *T* represents treatment failure, and they progress to the class *A* at the same rate *θ*_2_. Full-blown AIDS individuals *A* can also develop KS at rate *ϵ*_3_ > *ϵ*_2_ > *ϵ*_1_ due to weakened immune systems. Persons in the full-blown AIDS classes have additional AIDS-induced mortality *δ*_1_. Progression rate to acute KS for persons with full-blown AIDS is *τ*_3_, which we assume to be greater than *τ*_2_ and *τ*_1_. Individuals in both AIDS classes are also subject to a natural mortality rate of *μ*.

The force of infection *λ*_*h*_ depends on the probability of transmission per contact *β*, the proportion of infected individuals in each category (*I* and *I*_*k*_), the proportion of infected individuals in receipt of HAART (*T*), and the pre-AIDS classes (*P* and *P*_*k*_). Individuals in receipt of HAART have reduced viral load [20] and are therefore assumed to be less infectious relative to infectives not in receipt of HAART. This reduced infectiousness is modeled by *γ*_1_ < 1. Coinfected infectives and pre-AIDS individuals (*I*_*k*_ and *P*_*k*_) have weaker immune systems and are therefore likely to carry higher viral loads than their counterparts in the (*I* and *P*) classes. This added infectiousness is modeled by *γ*_2_ > 1 and *γ*_4_ > 1. Pre-AIDS infectives *A* are more infectious because of increased viral load, and this increased infectiousness is modeled by *γ*_3_ where *γ*_1_ < 1 < *γ*_2_ < *γ*_3_ < *γ*_4_. Persons with full-blown AIDS exhibit symptoms related to HIV and therefore are assumed to be noninteracting. The total sexually active variable population at time *t* is given by *N*(*t*) = *S*(*t*) + *I*(*t*) + *I*_*k*_(*t*) + *P*(*t*) + *P*_*k*_(*t*) + *T*(*t*). Assuming homogeneous mixing, the time dependent force of infection for HIV is given by

(1) $$\begin{array}{c} \lambda_{h}=\beta \left[\frac{\gamma_{1}T+I+\gamma_{2}I_{k}+\gamma_{3}P+\gamma_{4}P_{k}}{S+T+I+I_{k}+P\left(t\right)+P_{k}}\right]. \end{array}$$

**Figure 1:**
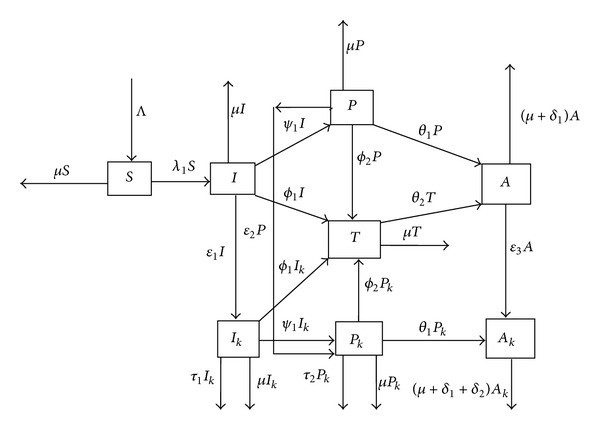
Model flow diagram.

The model flow diagram depicting this biological system is illustrated in Figure 1. 

The above assumptions and formulations lead us to this nonlinear system of differential equations:

(2) S′(t)=Λ−(λh+μ)S(t),I′(t)=λhS(t)−(ϵ1+μ+ψ1+ϕ1)I(t),Ik′(t)=ϵ1I(t)−(μ+ψ1+ϕ1+τ1)Ik(t),P′(t)=ψ1I(t)−(ϕ2+ϵ2+θ1+μ)P(t),Pk′(t)=ϵ2P(t)+ψ1Ik−(ϕ2+θ1+μ+τ2)Pk(t),T′(t)=ϕ1(I(t)+Ik(t))+ϕ2(P(t)+Pk(t))−(θ2+μ)T(t),A′(t)=θ1P(t)+θ2T(t)−(ϵ3+δ1+μ)A(t),Ak′(t)=θ1Pk(t)+ϵ3A(t)−(δ1+δ2+μ)Ak(t).

All parameters for the model system (2) are assumed to be nonnegative for all time *t* > 0. 

## 3. Basic Properties 

### 3.1. Positivity and Boundedness of Solutions

We denote by *R*_+_^8^ the set of points *x*_*t*_ = (*x*_1_, *x*_2_, *x*_3_, *x*_4_, *x*_5_, *x*_6_, *x*_7_, *x*_8_) in *R* ^8^ with positive coordinates and consider the system (2) with initial values

(3) $$\begin{array}{c} x^{0}=\left(x_{1}^{0},x_{2}^{0},x_{3}^{0},x_{4}^{0},x_{5}^{0},x_{6}^{0},x_{7}^{0},x_{8}^{0}\right). \end{array}$$

In this section, we prove the following lemma.

**Lemma**

The system (2) can be written as a system of differential inequalities

(4) $$\begin{array}{c} \frac{dx_{i}}{dt}\geq A_{i}x_{i}+\sum_{j}^{n}B_{ij}x_{j}+\epsilon \;\left(i=1,\ldots ,n\right), \end{array}$$

where

(5) $$\begin{array}{c} B_{ij}\geq 0,\;\epsilon \geq 0. \end{array}$$

If *x*_*i*_(0) ≥ *ϵ* then *x*_*i*_(*t*) ≥ 0 for all *t* > 0 and 1 ≤ *i* ≤ *n*.

Without loss of generality we may assume that *ϵ* > 0, since the case *ϵ* = 0 follows by approximating the system with a sequence *ϵ* = *ϵ*_*k*_ ↓ 0.

**Proof**

Suppose that the assertion *x*_*i*_(0) ≥ *ϵ* > 0, for 1 ≤ *i* ≤ *n*, is not true. Then there exists the smallest number *t*_0_ > 0, such that

(6) $$\begin{array}{c} x_{i}\left(t\right)>0\;\text{for}\;\;1\leq i\leq n,\;\;0\leq t\leq t_{0},\\ x_{i}\left(t_{0}\right)=0\;\text{for}\;\;\text{at}\;\;\text{least}\;\;\text{one}\;\;i,\;\;\text{say}\;\;i=i_{0}. \end{array}$$

Then *x*_*i*_0__ is a decreasing function at *t* = *t*_0_, so that

(7) $$\begin{array}{c} \frac{dx_{i_{0}}}{dt}\left(t_{0}\right)\leq 0. \end{array}$$

From the differential inequality (4) for *x*_*i*_0__(*t*) we get

(8) $$\begin{array}{c} \frac{dx_{i_{0}}}{dt}\left(t_{0}\right)\geq \sum_{j}^{n}B_{ij}x_{j}+\epsilon \geq \epsilon >0 \end{array}$$

which is a contradiction.

For the state variables in our model, we always take

(9) $$\begin{array}{c} S\left(0\right)\geq 0,\;\;I\left(0\right)\geq 0,\;\;I_{k}\left(0\right)\geq 0,\;\;P\left(0\right)\geq 0,\\ P_{k}\left(0\right)\geq 0,\;\;T\left(0\right)\geq 0,\;\;A\left(0\right)\geq 0,\;\;A_{k}\left(0\right)\geq 0. \end{array}$$

From Lemma 1 we conclude that

(10) $$\begin{array}{c} S\left(t\right)\geq 0,\;\;I\left(t\right)\geq 0,\;\;I_{k}\left(t\right)\geq 0,\;\;P\left(t\right)\geq 0,\\ P_{k}\left(t\right)\geq 0,\;\;T\left(t\right)\geq 0,\;\;A\left(t\right)\geq 0,\;\;A_{k}\left(t\right)\geq 0. \end{array}$$

Thus, in the region *R*_+_^8^ the model is epidemiologically and mathematically well posed and we can use it to study the dynamics of HIV-KS coinfection.

#### 3.1.1. Equilibrium States, Reproductive Number, and Stability Analysis

The model (2) possesses a disease-free equilibrium, *ξ*_0_, given by

(11) $$\begin{array}{c} \xi_{0}=\left\{S,I,I_{k},P,P_{k},T,A,A_{k}\right\}=\left\{\left(\frac{\Lambda }{\mu }\right),0,0,0,0,0,0,0\right\}, \end{array}$$

and at least one endemic equilibrium state whose existence is discussed in Section 3.3. Following Van Den Driessche and Watmough [21], the KS-induced reproductive number of system (2), *ℛ*_*k*_, is given by the spectral radius of the matrix *FV* ^−1^ where the matrices *F* and *V* are given by

(12) F=(βγ3βγ2βγ4βγ1β00000000000000000000),V=(−K10000ψ1−K2000ϵ10−K3000ϵ2ψ1−K40ϕ1ϕ2ϕ1ϕ2−K5),

where

(13) K1=(μ+ϵ1+ϕ1+ψ1),K2=(μ+ϵ2+θ1+ϕ2),K3=(μ+τ1+ϕ1+ψ1),K4=(μ+θ1+τ2+ϕ2),K5=(μ+θ2).

From (12) we have calculated the reproduction number

(14) $$\begin{array}{c} {ℛ}_{k}={ℛ}_{I}+{ℛ}_{I_{k}}+{ℛ}_{P}+{ℛ}_{P_{k}}+{ℛ}_{T}, \end{array}$$

where

(15) ℛI=β(μ+ϵ1+ϕ1+ψ1),ℛIk=βγ2ϵ1(μ+ϵ1+ϕ1+ψ1)(μ+τ1+ϕ1+ψ1),ℛP=βγ3ψ1(μ+ϵ2+θ1+ϕ2)(μ+ϵ1+ϕ1+ψ1),ℛPk= βγ4ψ1 ×(ϵ1(μ+ϵ2+θ1+ϕ2)+ϵ2(μ+τ1+ϕ1+ψ1))×((μ+ϵ2+θ1+ϕ2)(μ+θ1+τ2+ϕ2)   ×(μ+ϵ1+ϕ1+ψ1)(μ+τ1+ϕ1+ψ1))−1,ℛT=ℛTI+ℛT2+ℛT3+ℛT4+ℛT5,ℛT1=βγ1ϕ1(μ+θ2)(μ+ϵ1+ϕ1+ψ1),ℛT2=βγ1ϕ2ψ1(μ+θ2)(μ+ϵ2+θ1+ϕ2)(μ+ϵ1+ϕ1+ψ1),ℛT3=βγ1ϵ1ϕ1(μ+θ2)(μ+ϵ1+ϕ1+ψ1)(μ+τ1+ϕ1+ψ1),ℛT4=βγ1ϵ1ϕ2ψ1×((μ+θ2)(μ+θ1+τ2+ϕ2)   ×(μ+ϵ1+ϕ1+ψ1)(μ+τ1+ϕ1+ψ1))−1,ℛT5=βγ1ϵ2ϕ2ψ1×((μ+θ2)(μ+ϵ2+θ1+ϕ2)   ×(μ+θ1+τ2+ϕ2)(μ+ϵ1+ϕ1+ψ1))−1.

This number *ℛ*_*k*_ is a threshold such that if *ℛ*_*k*_ < 1 the disease clears from the population. If *ℛ*_*k*_ > 1 the steady state *ξ*_0_ becomes unstable and the disease establishes itself into the population. This number is comprehensively analyzed further in Section 3.4 to reveal the impact of treatment.

### 3.2. Global Stability of the Disease-Free Equilibrium

If *ℛ*_*k*_ < 1 then fixed point *ℰ*_0_ is locally asymptotically stable. We now determine conditions which guarantee global asymptotic stability of the disease-free state [22]. Rewrite model system (2) as

(16) $$\begin{array}{c} \frac{dX}{dt}=F\left(x,Z\right),\\ \frac{dZ}{dt}=G\left(X,Z\right),\;\;G\left(x,0\right)=0, \end{array}$$

where *X* ∈ ℝ^*m*^ denotes the number of uninfected individuals and *Z* ∈ ℝ^*n*^ denotes the number of infected individuals including those latently infected and those who are infectious. The disease-free equilibrium state can now be written as

(17) $$\begin{array}{c} U_{0}=\left(X^{*},0\right), \end{array}$$

where X* = ((Λ/*μ*), 0,0, 0,0, 0,0, 0,0). To guarantee global stability, the following conditions must be satisfied:

- for *dX*/*dt* = *F*(*X*, 0), X* is globally asymptotically stable;
- $G(X,Z)=\text{A}Z-\hat{G}(X,Z),\hat{G}(X,Z)\geq 0$ for (*X*, *Z*) ∈ *Ω*;
- *A* = *D*_*Z*_ *G*(X*, 0) is an *M*-matrix (the off diagonal elements of *A* are nonnegative) and *Ω* is the region where the model makes biological sense.

**Lemma 2**

The fixed point *U*_0_ = (X*, 0) is a globally asymptotically stable point of (2) provided that *ℛ*_*k*_ < 1 and conditions stated above are satisfied.

**Proof**

Consider

(18) $$\begin{array}{c} F\left(X,0\right)=\left(\begin{array}{c} \Lambda -\mu S_{f}\\ 0\\ 0\\ 0\\ 0 \end{array}\right),\\ G\left(X,Z\right)=AZ-\hat{G}\left(X,Z\right),\\ A=\left(\begin{array}{ccccc} \beta -K_{1} & \gamma_{3}\beta & \gamma 2\beta & \gamma_{4}\beta & \gamma_{1}\beta \\ \psi_{1} & -K_{2} & 0 & 0 & 0\\ \epsilon_{1} & 0 & -K_{3} & 0 & 0\\ 0 & \epsilon_{2} & \psi_{1} & -K_{4} & 0\\ \phi_{1} & \phi_{2} & \phi_{1} & \phi_{2} & -K_{5} \end{array}\right). \end{array}$$

Then

(19) G^(X,Z)=β(γ1T+Im+γ2Ik+γ3P+γ4Pk)×((1−SS+I+Ik+P+Pk)0000).

Since 0 ≤ *S* ≤ *S* + *I* + *I*_*k*_ + *P* + *P*_*k*_, it is clear that $\hat{G}(X,Z)\geq 0$ and *A* is an *M*-matrix whenever its spectral radius is less than unity. Then, by Lemma 2, *ξ*_0_ is a globally asymptotically stable equilibrium of (2).

The biological interpretation of Lemma 2 is that this population is observing the one partner policy or abstinence from unprotected sex resulting in HIV being eliminated from the population regardless of the size of the initial subpopulations [23].

### 3.3. Existence of Endemic Equilibria and Bifurcation Analysis

 In this section following the approach in [7], we establish conditions for the existence of endemic equilibria and investigate their stability. Denote the arbitrary endemic equilibrium of model (2) by ℰ* where

(20) $$\begin{array}{c} {ℰ}^{*}=\left(S^{*},I^{*},I_{k}^{*},T^{*}P^{*},P^{*},A^{*},A_{k}^{*}\right). \end{array}$$

Let

(21) $$\begin{array}{c} \lambda_{h}^{*}=\beta \left[\frac{\gamma_{1}T^{*}+I^{*}+\gamma_{2}I_{k}^{*}+\gamma_{3}P^{*}+\gamma_{4}P_{k}^{*}}{S^{*}+T^{*}+I^{*}+I_{k}^{*}+P^{*}\left(t\right)+P_{k}^{*}}\right], \end{array}$$

be the force of infection. By setting the right hand side of model (2) to zero, we obtain, in terms of the force of infection,

(22) $$\begin{array}{c} S^{*}=\frac{\Lambda }{\lambda_{h}^{*}+\mu },\;\;I^{*}=\frac{\lambda_{h}^{*}}{K_{1}},\;\;I_{k}^{*}=\frac{\epsilon_{1}I^{*}}{K_{2}},\\ T^{*}=\frac{\psi_{1}I^{*}}{K_{3}},\;\;P^{*}=\frac{\left(\psi_{1}I_{k}^{*}+\epsilon_{2}P^{*}\right)}{K_{4}},\\ A^{*}=\frac{\left(\theta_{1}I^{*}+\theta_{2}T^{*}\right)}{\left(\epsilon_{2}+\mu +\delta_{1}\right)},\;\;A_{k}=\frac{\left(\theta_{1}I_{k}+\epsilon_{3}A_{k}\right)}{\left(\mu +\delta_{1}+\delta_{2}\right)}. \end{array}$$

Substituting the values of S*, I*, *I*_k_*, T*P*, P*, A*, and *A*_k_* into (21) we obtain the polynomial

(23) $$\begin{array}{c} a_{0}\lambda_{h}^{2}+K_{1}\lambda_{h}=0, \end{array}$$

where

(24) a0=K2K4(K3+ϵ1)(μ+θ2+ϕ1)+(K2ϵ1+K3(K4+ϵ2))(μ+θ2+ϕ2)ψ1,a1=−βγ1(K3+ϵ1+(K2ϵ1+K3(K4+ϵ2))ϕ2ψ1)+(μ+θ2)(−βK3(K4γ3+γ4ϵ2)ψ1−K2(β−K1)K3K4 +βϵ1(K4γ2+γ4ψ1)).  

Equation (23) has two solutions given by

(25) λh0=0,λh1=K1K2K3K4(K2K4(ℛK−1)K6K8+ℛKγ1K9ϕ1+(ℛK−1)K10K8+ℛKγ1K7ϕ2)ψ1×((K2K4K9(K8+ϕ1)+K7(K8+ϕ2)ψ1)   ×(K5ψ1+K2(K4K6+γ4ϵ1ψ1)))−1,

where

(26) $$\begin{array}{c} K_{5}=K_{3}\left(K_{4}\gamma_{3}+\gamma_{4}\epsilon_{2}\right),\;K_{6}=\left(K_{3}+\gamma_{2}\epsilon_{1}\right),\\ K_{8}=\left(\mu +\theta_{2}\right),\;K_{9}=\left(K_{3}+\epsilon_{1}\right),\;K_{10}=\left(K_{2}\gamma_{4}\epsilon_{1}+K_{5}\right). \end{array}$$

From the solution *λ*_*h*1_, the condition *ℛ*_*K*_ > 1 is necessary for existence of the nontrivial endemic equilibrium and we summarize this as follows.

**Lemma 3**

The model (2) has a unique endemic equilibrium whenever *ℛ*_*k*_ > 1.

Having proved the existence of the endemic equilibrium point we now investigate its stability using the Centre Manifold Theory [24] as described by Castillo-Chavez and Song [25]. Consider the following general system of ordinary differential equations with a parameter *ϕ*

(27) $$\begin{array}{c} \frac{dx}{dt}=f\left(x,\phi \right),\;f:{\mathbb{R}}^{n}\times \mathbb{R}\to \mathbb{R},\;\;f\in C^{2}\left(\mathbb{R}\times \mathbb{R}\right). \end{array}$$

Without loss of generality, it is assumed that 0 is an equilibrium for system (27) for all values of the parameter *ϕ*, (i.e., *f*(*ϕ*, 0) ≡ 0 for all *ϕ*).

Assume the following.

(A1): *A* = *D*_*x*_ *f*(0,0) = ((∂*f*_*i*_/∂*x*_*j*_)(0,0)) is the linearise matrix of system (27) around the equilibrium 0 with *ϕ* evaluated at 0. Zero is a simple eigenvalue of *A* and all other eigenvalues of *A* have negative real parts.
(A2): Matrix *A* has a nonnegative right eigenvector *w* and a left eigenvector *v* corresponding to the zero eigenvalue.

Let *f*_*k*_ be the *k*th component of *f* and

(28) $$\begin{array}{c} a=\sum_{k,i,j=1}^{n}v_{k}w_{i}w_{j}\frac{\partial^{2}f_{k}}{\partial x_{i}\partial x_{j}}\left(0,0\right),\\ b=\sum_{k,i=1}^{n}v_{k}w_{i}\frac{\partial^{2}f_{k}}{\partial x_{i}\partial \phi }\left(0,0\right). \end{array}$$

The local dynamics of system (27) around 0 are totally determined by *a* and *b*.

- *a* > 0, *b* > 0. When *ϕ* < 0 with |*ϕ*| ≪ 1, 0 is locally asymptotically stable and there exists a positive unstable equilibrium; when 0 < *ϕ* ≪ 1, 0 is unstable and there exists a negative and locally asymptotically stable equilibrium.
- *a* < 0, *b* < 0. When *ϕ* < 0 with |*ϕ*| ≪ 1, 0 is unstable; when 0 < *ϕ* ≪ 1, 0 is locally asymptotically stable, and there exists a positive unstable equilibrium.
- *a* > 0, *b* < 0. When *ϕ* < 0 with |*ϕ*| ≪ 1, 0 is unstable, and there exists a locally asymptotically stable negative equilibrium; when 0 < *ϕ* ≪ 1, 0 is stable, and a positive unstable equilibrium appears.
- *a* < 0, *b* > 0. When *ϕ* changes from negative to positive, 0 changes its stability from stable to unstable. Correspondingly, a negative unstable equilibrium becomes positive and locally asymptotically stable. Particularly, if *a* > 0 and *b* > 0, then, a backward bifurcation occurs at *ϕ* = 0.

We make the following change of variables: *S* = *x*_1_, *I* = *x*_2_, *I*_*k*_ = *x*_3_, *P* = *x*_4_, *P*_*k*_ = *x*_5_, *T* = *x*_6_, *A* = *x*_7_, and *A*_*k*_ = *x*_8_ so that *N* = ∑_*n*=1_^8^ *x*_*n*_. We now use the vector notation *X* = (*x*_1_, *x*_2_, *x*_3_, *x*_4_, *x*_5_, *x*_6_, *x*_7_, *x*_8_)^*T*^. Then, model system (2) can be written in the form *dX*/*dt* = *F* = (*f*_1_, *f*_2_, *f*_3_, *f*_4_, *f*_5_, *f*_6_, *f*_7_, *f*_8_)^*T*^, such that

(29) x1′(t)=f1=Λ−(λh+μ)x1(t),x2′(t)=f2=λhx1(t)−(ϵ1+μ+ψ1+ϕ1)x2(t),x3′(t)=f3=ϵ1x2(t)−(μ+ψ1+ϕ1+τ1)x3(t),x4′(t)=f4=ψ1x2(t)−(ϕ2+ϵ2+θ1+μ)x4(t),x5′(t)=f5=ϵ2x4(t)+ψ1x3−(ϕ2+θ1+μ+τ2)x5(t),x6′(t)=f6=ϕ1(x2(t)+x3(t))+ϕ2(x4(t)+x5(t))−(θ2+μ)x6(t),x7′(t)=f7=θ1x4(t)+θ2x6(t)−(ϵ3+δ1+μ)x7(t),x8′(t)=f8=θ1x5(t)+ϵ3x7(t)−(δ1+δ2+μ)x8(t),

where

(30) $$\begin{array}{c} \lambda_{h}=\frac{\gamma_{1}x_{6}+x_{2}+\gamma_{2}x_{3}+\gamma_{3}x_{4}+\gamma_{4}x_{5}}{x_{1}+x_{2}+x_{3}+x_{4}+x_{5}+x_{6}}. \end{array}$$

The Jacobian matrix of system (29) at the disease-free equilibrium is given by

(31) $$\begin{array}{c} J\left(\xi_{0}\right)=\left(\begin{array}{ccccc} \beta -K_{1} & \gamma_{3}\beta & \gamma 2\beta & \gamma_{4}\beta & \gamma_{1}\beta \\ \psi_{1} & -K_{2} & 0 & 0 & 0\\ \epsilon_{1} & 0 & -K_{3} & 0 & 0\\ 0 & \epsilon_{2} & \psi_{1} & -K_{4} & 0\\ \phi_{1} & \phi_{2} & \phi_{1} & \phi_{2} & -K_{5} \end{array}\right), \end{array}$$

from which it can be shown that *ℛ*_*k*_ is the same as in (14). If β* is taken as a bifurcation point and if we consider the case *ℛ*_1_ = 1 and solve for β* = *β* gives

(32) $$\begin{array}{c} \beta^{*}=\frac{1}{{ℛ}_{k}}. \end{array}$$

The linearised system of the transformed equations (29) at β* has a simple zero eigenvalue. Hence, the Centre Manifold Theory [24] can be used to analyse the dynamics of system (29) near β*. It can be shown that the Jacobian of (29) at β* has a right eigenvector associated with the zero eigenvalue given by *u* = [*u*_1_, *u*_2_, *u*_3_, *u*_4_, *u*_5_, *u*_6_, *u*_7_, *u*_8_]^*T*^, where

(33) $$\begin{array}{c} u_{2}=u_{2}>0,\;u_{3}=\frac{u_{2}\psi_{1}}{K_{2}}>0,\\ u_{4}=\frac{u_{2}\epsilon_{1}}{K_{3}}>0,\;u_{6}=\frac{u_{2}\phi_{1}+u_{4}\phi_{1}+\left(u_{3}+u_{5}\right)\phi_{2}}{K_{5}}>0,\\ u_{7}=\frac{u_{2}\theta_{1}+u_{6}\theta_{2}}{K_{6}}>0,\;u_{8}=\frac{u_{7}\epsilon_{3}+u_{4}\theta_{1}}{K_{7}}>0,\\ u_{1}=-\frac{\beta \left(u_{2}+u_{6}\gamma_{1}+u_{4}\gamma_{2}+u_{3}\gamma_{3}+u_{5}\gamma_{4}\right)}{\mu }<0. \end{array}$$

The left eigenvector of *J*(*ξ*_0_) associated with the zero eigenvalue at *β* = β* is given by *v* = [*v*_1_, *v*_2_, *v*_3_, *v*_4_, *v*_5_, *v*_6_, *v*_7_, *v*_8_] where

(34) $$\begin{array}{c} v_{1}=v_{7}=v_{8}=0,\;v_{2}=v_{2}>0,\\ v_{3}=\frac{\beta v_{2}\gamma_{3}+v_{5}\epsilon_{2}+v_{6}\phi_{2}}{K_{2}}>0,\;v_{4}=\frac{\beta v_{2}\gamma_{2}+v_{6}\phi_{1}+v_{5}\psi_{1}}{K_{3}},\\ v_{5}=\frac{\beta v_{2}\gamma_{4}+v_{6}\phi_{2}}{K_{4}},\;v_{6}=\frac{\beta v_{2}\gamma_{1}}{K_{5}}. \end{array}$$

For the model system (2), the associated nonzero partial derivatives of *F* at the disease-free equilibrium are given by

(35) $$\begin{array}{c} \frac{\partial f_{2}^{2}}{\partial x_{2}\partial x_{2}}=-\frac{2\beta \mu }{\Lambda },\;\frac{\partial f_{2}^{2}}{\partial x_{2}\partial x_{3}}=\frac{\partial f_{2}^{2}}{\partial x_{2}\partial x_{3}}=-\frac{\beta \mu \left(1+\gamma_{2}\right)}{\Lambda },\\ \frac{\partial f_{2}^{2}}{\partial x_{2}\partial x_{4}}=\frac{\partial f_{2}^{2}}{\partial x_{4}\partial x_{2}}=-\frac{\beta \mu \left(1+\gamma_{3}\right)}{\Lambda },\\ \frac{\partial f_{2}^{2}}{\partial x_{2}\partial x_{5}}=-\frac{\partial f_{2}^{2}}{\partial x_{5}\partial x_{2}}=\frac{\beta \mu \left(1+\gamma_{4}\right)}{\Lambda },\\ \frac{\partial f_{2}^{2}}{\partial x_{2}\partial x_{6}}=\frac{\partial f_{2}^{2}}{\partial x_{6}\partial x_{2}}=-\frac{\beta \mu u_{6}\left(1+\gamma_{1}\right)}{\Lambda },\\ \frac{\partial f_{2}^{2}}{\partial x_{3}\partial x_{3}}=-\frac{2\beta \mu \gamma_{2}}{\Lambda },\;\frac{\partial f_{2}^{2}}{\partial x_{3}\partial x_{4}}=\frac{\partial f_{2}^{2}}{\partial x_{4}\partial x_{3}}=-\frac{\beta \mu \left(\gamma_{2}+\gamma_{3}\right)}{\Lambda },\\ \frac{\partial f_{2}^{2}}{\partial x_{3}\partial x_{5}}=\frac{\partial f_{2}^{2}}{\partial x_{5}\partial x_{3}}=-\frac{\beta \mu \left(\gamma_{2}+\gamma_{4}\right)}{\Lambda },\\ \frac{\partial f_{2}^{2}}{\partial x_{3}\partial x_{6}}=\frac{\partial f_{2}^{2}}{\partial x_{6}\partial x_{3}}=\frac{\beta \mu \left(\gamma_{1}+\gamma_{2}\right)}{\Lambda },\\ \frac{\partial f_{2}^{2}}{\partial x_{4}\partial x_{4}}=-\frac{2\beta \mu \gamma_{3}}{\Lambda },\;\frac{\partial f_{2}^{2}}{\partial x_{4}\partial x_{5}}=\frac{\partial f_{2}^{2}}{\partial x_{5}\partial x_{4}}=\frac{\beta \mu \left(\gamma_{3}+\gamma_{4}\right)}{\Lambda },\\ \frac{\partial f_{2}^{2}}{\partial x_{4}\partial x_{6}}=\frac{\partial f_{2}^{2}}{\partial x_{6}\partial x_{4}}=-\frac{\beta \mu \left(\gamma_{1}+\gamma_{3}\right)}{\Lambda },\\ \frac{\partial f_{2}^{2}}{\partial x_{5}\partial x_{5}}=\frac{2\beta \mu \gamma_{4}}{\Lambda },\;\frac{\partial f_{2}^{2}}{\partial x_{5}\partial x_{6}}=\frac{\partial f_{2}^{2}}{\partial x_{6}\partial x_{5}}=\frac{\beta \mu \left(\gamma_{1}+\gamma_{4}\right)}{\Lambda },\\ \frac{\partial f_{2}^{2}}{\partial x_{6}\partial x_{6}}=\frac{2\beta \mu \gamma_{1}}{\Lambda }. \end{array}$$

From (28), it follows that

(36) a=−1ΛK22K32K42K52×(2βμu22v2(K2K4(K3+ϵ1)(K5+ϕ1)+(K2ϵ1+K3(K4+ϵ2))(K5+ϕ2)ψ1)×(K3(K5(a4γ2+γ4ϵ2)+γ1(K4+ϵ2)ϕ2)ψ1    +K2(K4(K5(K3+γ3ϵ1)+γ1(K3+ϵ1)ϕ1)        +ϵ1(K5γ4+γ1ϕ2)ψ1)))<0.

For the sign of *b*, it is associated with the nonvanishing partial derivatives of *F*,

(37) $$\begin{array}{c} \frac{\partial^{2}f_{2}}{\partial_{x_{2}}\partial_{\beta_{m}}}=1,\;\;\frac{\partial^{2}f_{2}}{\partial_{x_{3}}\partial_{\beta_{m}}}=\gamma_{2},\;\;\frac{\partial^{2}f_{2}}{\partial_{x_{4}}\partial_{\beta_{m}}}=\gamma_{3},\\ \frac{\partial^{2}f_{2}}{\partial_{x_{5}}\partial_{\beta_{m}}}=\gamma_{4},\;\;\frac{\partial^{2}f_{2}}{\partial_{x_{6}}\partial_{\beta_{m}}}=\gamma_{1}. \end{array}$$

From (28), it follows that

(38) b=v2 [u2∂2f2∂x2∂βm+u3∂2f2∂x3∂βm+u4∂2f2∂x4∂βm  +u5∂2f2∂x5∂βm+u6∂2f2∂x6∂βm]>0.

By Theorem 11 in [25] we establish result in Lemma 4.

**Lemma**

Since *a* < 0, then model system (2) has a forward transcritical bifurcation and a unique locally stable endemic equilibrium (ℰ*) guaranteed by Lemma 3 exists for *ℛ*_*k*_ > 1 but sufficiently close to 1.

### 3.4. Analysis of the Reproductive Number *ℛ*_*k*_

To analyze the coinfection dynamics of KS and HIV, we investigate the KS-induced reproductive number in (14). In (14), we note that the partial reproductive numbers *ℛ*_*I*_, *ℛ*_*I*_*K*__, *ℛ*_*P*_, *ℛ*_*P*_*K*__, and *ℛ*_*T*_ represent the contribution to the reproduction number *ℛ*_*k*_ from the following classes of infectives HIV only (*I*), coinfected (*I*_*k*_), Pre-AIDS (*P*), coinfected pre-AIDS (*P*_*k*_), and those receiving HAART treatment (*T*). The partial reproductive number *ℛ*_*T*_ representing the contribution of groups in receipt of ART is split into *ℛ*_*T*_1__, *ℛ*_*T*_2__, *ℛ*_*T*_3__, *ℛ*_*T*_4__, and *ℛ*_*T*_5__ and represents the contribution of HIV infectives, infectives dually infected with KS, Pre-AIDS infectives, and Pre-AIDS infectives dually infected with KS, respectively, who receive anti-retroviral therapy. 

If we define *ℛ*_0_ to be the basic HIV reproductive number in the absence of KS and HIV treatment and set parameters related to KS and treatment to zero in (14) we obtain

(39) $$\begin{array}{c} {ℛ}_{K\;|\;\epsilon_{1}=\epsilon_{2}=\phi_{1}=\phi_{2}=0}={ℛ}_{0}={ℛ}_{I}+{ℛ}_{P}. \end{array}$$

If we define the partial reproductive number without treatment to be *ℛ*_*NT*_ and set parameters related to treatment to zero then we obtain

(40) ℛK ∣ ϕ1=ϕ2=0=ℛNT,=ℛI+ℛIK+ℛP+ℛPK>ℛ0.

**Figure 2:**
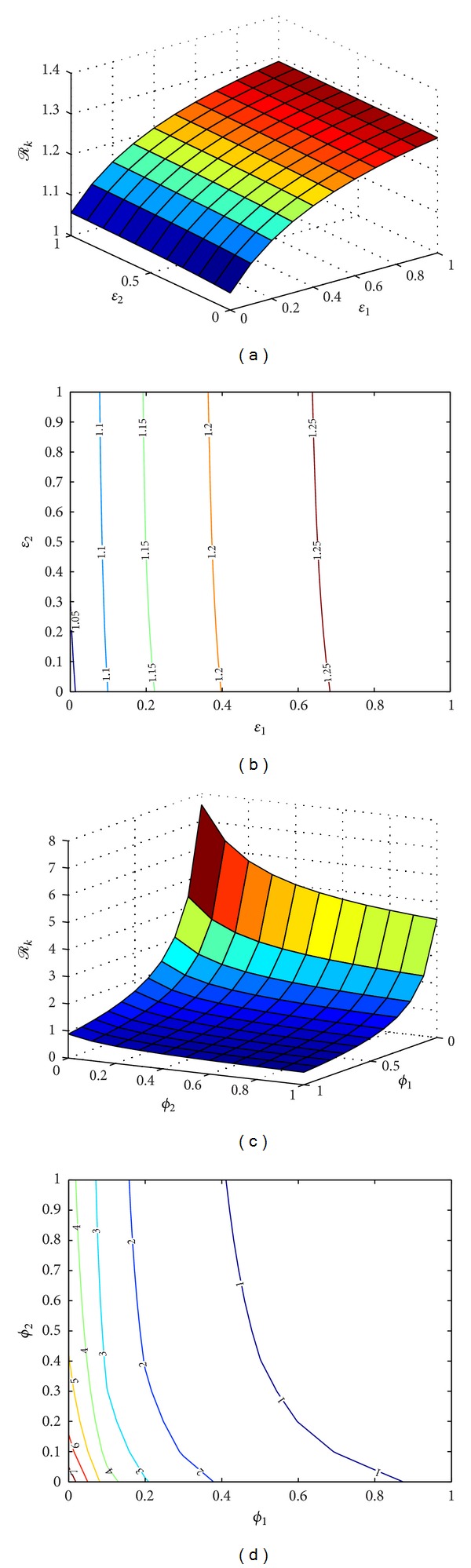
(a) 3D plot of *ℛ*_*K*_ against the rates of KS manifestation for infected and pre-AIDS classes for variable values of *ϵ*_1_ and *ϵ*_2_, all other parameters constant as in Table 1, (b) contour plot map of *ℛ*_*K*_ against *ϵ*_1_ and *ϵ*_2_, all other parameters constant as in Table 1, (c) 3D plot of *ℛ*_*K*_ against the rates of ART administration for infected and pre-AIDS classes for variable values of *ϕ*_1_ and *ϕ*_2_, all other parameters constant as in Table 1, and (d) contour plot map of *ℛ*_*K*_ against *ϕ*_1_ and *ϕ*_2_, all other parameters constant as in Table 1.

If *ℛ*_0_ > 1, then not providing treatment to all infective classes worsens the HIV in this population. Note that even if *ℛ*_0_ < 1, the option not to provide treatment to all infective classes could increase the reproduction number above 1. Treatment to all infective classes is necessary to effectively control the HIV disease spread. Using sensitivity analysis on *ℛ*_*K*_, we have reinforced some of these findings as shown in Figure 2. 

From Figures 2(a) and 2(b) wem note that an increase in the rate of infected people who develop KS will lead to an increase in *ℛ*_*K*_ and consequently an increase in the HIV epidemic. However, an increase in the rate of progress to dual infection for people in the pre-AIDS class will result in a marginal increase of *ℛ*_*K*_ and hence the HIV epidemic.

Figures 2(c) and 2(d) show that treatment of infected individuals and dually infected individuals will have a much higher positive impact on *ℛ*_*K*_ and the epidemic than treatment at the pre-AIDS stage and this leads us to conclude that early administration of HAART on HIV infectives to curtail the growth of opportunistic infections, such as KS, will have a more positive impact than delayed therapy at the pre-AIDS stage. 

## 4. Numerical Simulations

Using the R programming environment, we ran numerical simulations of the model. The following data were input as initial conditions:

(41) $$\begin{array}{c} N_{0}=\left[I,I_{k},P,P_{k},T,A,A_{k},S\right]=\left[1,0,0,0,0,0,0,800\right]. \end{array}$$

Parameter values used in the numerical simulations of model system (2) are shown in Table 1. HIV/KS coinfection study is still in its infancy. A number of numeric values for the parameters shown in Table 1 are reasonable estimates. Some of the parameters were estimated by modifying baseline values from published literature and these are denoted with an asterisk in Table 1.

Our value for the rate of HIV transmission is the average of the minimum (0.011) and maximum values (0.95) for the same parameter in Baggaley et al. [5] and Boily et al. [6]. We assumed that a death rate due to AIDS and KS coinfection would be 0.4; in our model, *δ*_1_ and *δ*_2_ are summed. Our recruitment rate is calculated by incorporating an annual recruitment rate (0.029) from Malunguza et al. [7]. In this study, we take the population of sub-Sahara Africa to be 767 million as in Mukandawire et al. [16] and Barley et al. [13].

**Figure 3:**
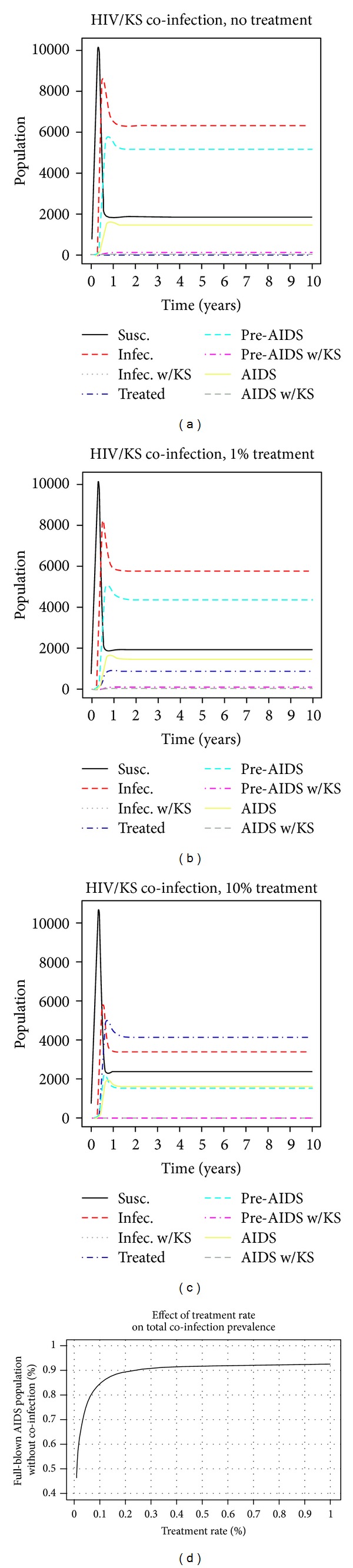
Epidemic curves for our model with the following treatment scenarios: (a) no treatment (*ϕ*_1_ = *ϕ*_2_ = 0), (b) 1% treatment level (*ϕ*_1_ = *ϕ*_2_ = 0.01), and (c) 10% treatment level (*ϕ*_1_ = *ϕ*_2_ = 0), (d) treatment rate versus AIDS population without coinfection. A treatment level of 10% is sufficient at minimizing endemic populations in all the infective, untreated classes at the equilibrium.

Figure 3(a) shows that in the absence of treatment, the number of individuals with the AIDS/KS coinfection exceeds the number of those with just AIDS; that is, 54.0% of the total AIDS population has KS by the end of the model. Merely affecting a treatment rate of 1%, is enough to reverse this relation as depicted in Figure 3(b) where 61.7% of all AIDS patients are devoid of KS. If the treatment rate is increased to 10%, the AIDS population without the coinfection increases to 85.7% as depicted in Figure 3(c). Using parameter values in Table 1 causes the model to tend towards some endemic equilibrium over time. In other words, the DFE is unstable, and so we expect *ℛ*_0_ > 1. In fact, carrying out these computations with *ϕ*_1_ = *ϕ*_2_ = 0.1, we obtain *ℛ*_0_ ≈ 4.55. This value is slightly higher than estimated values of *ℛ*_0_ for HIV/AIDS in European countries, which is consistent with our expectations [26].

Figure 3(d) provides an interesting insight into the predictions of our model. By providing treatment to 100% of all infectives and pre-AIDS individuals the model fails to eradicate the coinfection, and approximately an 8% prevalence of the coinfection among the full-blown AIDS cohort exists. This result means that we need to be cognisant of two objectives, namely, that we should have benchmark target goals in the provision of treatment and that work should be done to decrease the number of patients entering the pre-AIDS coinfection class. It is entirely unrealistic to presume that the coinfection can be eliminated entirely based on providing treatment to infectives and pre-AIDS individuals alone. In fact, Figure 3(d) shows that providing a treatment rate of *ϕ*_1_ = *ϕ*_2_ = 0.2 results in about 90% of the AIDS population losing the coinfection. Providing any further increases to *ϕ*_1_ and *ϕ*_2_ results in only a 2% increase.

 Our model makes no attempt to consider the cost associated with providing the kind of treatment we have discussed. However, it is noteworthy to consider that the gains associated with providing treatment to any more than 20% of all infectives and pre-AIDS individuals are minimal. It is clear that there is an 8% gap between where we would like to see the percentage of the full-blown AIDS cohort without coinfection and where our model currently estimates that percentage to be. Assuming that it is unrealistic to completely eradicate the coinfection, steps should be taken to shift the curve in Figure 3(d) closer to 100%. We believe that a good way to do this is by screening patients for the coinfection once the HIV infection is first diagnosed. In general, this will serve to decrease the total number of patients who become pre-AIDS with the coinfection and, in turn, this will decrease the total number of individuals presenting with the coinfection.

## 5. Discussion

In numerous HIV-positive cohorts, susceptibility to KS development is incredibly high. Recent findings that antiretroviral treatment for HIV clinical symptoms can reverse KS in most patients suggest that the only obstacle in preventing KS-related complications (or even deaths) is one's ability to access the treatment itself. This is especially the case in sub-Saharan Africa, where KS prevalence is higher than anywhere else in the world, yet access to proper treatment remains low. We have shown that providing treatment to just 10% of HIV-infected individuals, regardless of their KS status, can offer a significant reduction in the overall number of individuals who end up with full-blown AIDS and KS. Certainly, we expect that, as we increase the treatment rate, the disparity will become more and more favorable. However, our results only apply to situations in which individuals develop KS on their own—that is, in the absence of interaction with HHV-8-infected individuals. While such a generalization may be perfectly valid in sub-Saharan Africa, it will not apply to other regions where HHV-8 needs to be acquired sexually or through saliva. In other words, this model is very specific to the sub-Saharan region. 

In the future, we hope to extend this model to consider all modes of HHV-8 transmission. One way to do this is by introducing separate classes of HHV-8 infectious individuals. This will increase the overall reach of the model and hopefully provide a positive influence on policy making in areas affected by KS. For now, supporting KS education and awareness needs to become as important as providing universal access to antiretroviral treatment for HIV patients. Most southern African countries already have programs in place to encourage HIV screening; this is the perfect time to also test for HHV-8 and provide KS counseling. Regardless of how long the HIV/AIDS epidemic lasts in Africa, there is no reason people should have to fight KS as well.

## Figures and Tables

**Table 1:** Parameter values and their estimates.

Parameter	Symbol	Value	Source
HIV rate of transmission	*β*	0.4801*	Baggaley et al. [5],
			Boily et al. [6]
Death due to AIDS	*δ*_1_	0.333	Malunguza et al. [7],
			Mukandavire et al. [8]
Death due to AIDS with KS	*δ*_2_	0.067*	Malunguza et al. [7],
			Mukandavire et al. [8]
Rate of KS acquisition among HIV-infected cohort	*ϵ*_1_	0.001	Assumed
Rate of KS acquisition among pre-AIDS cohort	*ϵ*_2_	0.002	Assumed
Rate of KS acquisition among AIDS cohort	*ϵ*_3_	0.003	Assumed
Relative HIV infectiousness of an HIV-infected individual	*γ*_1_	0.8	Assumed
Relative HIV infectiousness of a coinfected individual	*γ*_2_	1.1	Assumed
Relative HIV infectiousness of a pre-AIDS individual	*γ*_3_	1.2	Assumed
Relative HIV infectiousness of an AIDS individual	*γ*_4_	1.3	Assumed
Recruitment rate of sexually mature individuals	Λ	800*	Barley et al. [9],
			Malunguza et al. [7]
			Mukandavire et al. [8, 10, 11]
Natural mortality rate	*μ*	0.02	Mukandavire et al. [8]
Treatment rate of infected and coinfected cohorts	*ϕ*_1_	Varies	Assumed
Treatment rate of pre-AIDS and pre-AIDS coinfected cohorts	*ϕ*_2_	Varies	Assumed
Natural progression to pre-AIDS	*ψ*_1_	0.01	Assumed
Rate of acute KS development in coinfected cohort	*τ*_1_	0.0001	Assumed
Rate of acute KS development in pre-AIDS coinfected cohort	*τ*_2_	0.0002	Assumed
Rate of acute KS development in AIDS coinfected cohort	*τ*_3_	0.0003	Assumed
Natural progression to AIDS	*θ*_1_	0.1	Mukandavire et al. [8, 10, 11]
Natural progression to AIDS after treatment	*θ*_2_	0.1	Assumed

*Denotes a parameter obtained by modifying a value in the given source.

## References

[1] Biotrin International HHV-8: questions and answers. http://www.diasorin.com/HHV8/FAQ.html.

[2] Foreman KE, Friborg J, Kong W-P (1997). Propagation of a human herpesvirus from AIDS-associated Kaposi’s sarcoma. *New England Journal of Medicine*.

[3] Dugdale DC, Vyas JM, Zieve D Kaposi’s sarcoma. http://www.ncbi.nlm.nih.gov/pubmedhealth/PMH0001682.

[4] Sitas F, Parkin M, Chirenje Z, Stein L, Mqoqi N, Wabinga H (2013). Cancers. *Disease and Mortality in Sub-Saharan Africa*.

[5] Baggaley RF, White RG, Boily MC (2008). Systematic review of orogenital HIV-1 transmission probabilities. *International Journal of Epidemiology*.

[6] Boily M-C, Baggaley RF, Wang L (2009). Heterosexual risk of HIV-1 infection per sexual act: systematic review and meta-analysis of observational studies. *The Lancet Infectious Diseases*.

[7] Malunguza NJ, Hove-Musekwa SD, Musuka G, Mukandavire Z (2012). Investigating al-cohol consumption as a risk factor for HIV transmission in heterosexual settings in sub-saharan african communities. *Bulletin of Mathematical Biology*.

[8] Mukandavire Z, Chiyaka C, Magombedze G, Musuka G, Malunguza NJ (2009). Assessing the effects of homosexuals and bisexuals on the intrinsic dynamics of HIV/AIDS in heterosexual settings. *Mathematical and Computer Modelling*.

[9] Barley K, Murillo D, Roudenko S, Tameru AM, Tatum S (2012). A mathematical model of HIV and malaria co-infection in sub-saharan Africa. *Journal of AIDS and Clinical Research*.

[10] Mukandavire Z, Garira W (2007). Sex-structured HIV/AIDS model to analyse the effects of condom use with application to Zimbabwe. *Journal of Mathematical Biology*.

[11] Mukandavire Z, Malunguza NJ, Chiyaka C, Musuka G, Tchuenche JM (2009). HIV/AIDS model assessing the effects of gender-inequality affecting women in african heterosexual settings. *International Journal of Biomathematics*.

[12] Lungu EM Stability of non-autonomous co-infection models. https://www.masamu.auburn.edu/ConMan/ConMan.

[13] Cohen T, Lipsitch M, Walensky RP, Murray M (2006). Beneficial and perverse effects of isoniazid preventive therapy for latent tuberculosis infection in HIV-tuberculosis coinfected populations. *Proceedings of the National Academy of Sciences of the United States of America*.

[14] Roeger L-IW, Feng Z, Castillo-Chavez C (2009). Modeling TB and HIV co-infections. *Mathematical Biosciences and Engineering*.

[15] Ramkissoon S, Mwambi HG, Matthews AP (2012). Modelling HIV and MTB co-infection including combined treatment strategies. *PLoS One*.

[16] Chiyaka C, Tchuenche JM, Garira W, Dube S (2008). A mathematical analysis of the effects of control strategies on the transmission dynamics of malaria. *Applied Mathematics and Computation*.

[17] Mukandavire Z, Gumel AB, Garira W, Tchuenche JM (2009). Mathematical analysis of a model for HIV-malaria co-infection. *Mathematical Biosciences and Engineering*.

[18] http://www.webmd.com/hiv-aids/guide/aids-hiv-opportunistic-infections-kaposis-sarcoma__.

[19] Von Roenn JH Treatment of HIV-associated kaposi sarcoma. http://hivinsite.ucsf.edu/InSite?page=kb-06-02-04.

[20] Wilson DP, Matthew GL, Grulich AE, Cooper DA, Kaldor JA Relation between HIV viral load and infectiousness: a model-based anal-ysis. *The Lancet*.

[21] Van Den Driessche P, Watmough J (2002). Reproduction numbers and sub-threshold endemic equilibria for compartmental models of disease transmission. *Mathematical Biosciences*.

[22] Castillo-Chavez C, Feng Z, Huang W On the computation of *ℛ*
_0_ and its role on global stability. http://math.la.asu.edu/chavez/2002/JB276.pdf.

[23] Sharomi O, Gumel AB (2009). Re-infection-induced backward bifurcation in the transmission dynamics of chlamydia trachomatis. *Journal of Mathematical Analysis and Applications*.

[24] Carr J (1981). *Applications Centre Manifold Theory*.

[25] Castillo-Chavez C, Song B (2004). Dynamical models of tuberculosis and their applications. *Mathematical Biosciences and Engineering*.

[26] Nishiura H (2010). Correcting the actual reproduction number: a simple method to estimate R0 from early epidemic growth data. *International Journal of Environmental Research and Public Health*.

